# Prevalence of Human Papillomavirus Infection Among Women Born in the 1990s vs the 1980s and Association With HPV Vaccination in the US

**DOI:** 10.1001/jamahealthforum.2022.2706

**Published:** 2022-08-19

**Authors:** Zahed Shahmoradi, Haluk Damgacioglu, Jane Montealegre, Elizabeth Y. Chiao, Keith Sigel, Kalyani Sonawane, Ashish A. Deshmukh

**Affiliations:** 1 Center for Health Services Research, Department of Management, Policy, and Community Health, The University of Texas Health Science Center School of Public Health, Houston; 2 Department of Pediatrics, Baylor College of Medicine, Houston, Texas; 3 Department of Epidemiology, The University of Texas MD Anderson Cancer Center, Houston; 4 Department of General Internal Medicine, Department of Medicine, Mount Sinai Icahn School of Medicine, New York, New York

## Abstract

This cross-sectional study examines rates of human papillomavirus infections among a cohort of vaccinated and unvaccinated women born in the 1990s.

## Introduction

In the US, human papillomavirus (HPV) vaccination was first recommended for routine use among girls and young women (9-26 years old) in 2006.^1^ Steady improvements in HPV vaccination coverage during the past decade may have translated into protective benefits among the contemporary birth cohort (ie, women born in the 1990s compared with the 1980s). To measure whether HPV vaccination has been associated with reduced infection rates among recently born vaccinated women (vaccine-mediated immunity) and unvaccinated women (herd protection), we compared HPV prevalence in the 1980s vs the 1990s birth cohorts and a prevaccination period vs a recent period.

## Methods

The institutional review board of The University of Texas Health Science Center deemed this study exempt from review and waived the requirement for informed consent because only publicly available data were used. We followed the Strengthening the Reporting of Observational Studies in Epidemiology (STROBE) reporting guideline.

This cross-sectional study analyzed data from 2 cycles (2005-2006 and 2015-2016) of the National Health and Nutritional Examination Survey (NHANES)—a stratified multistage probability sample of the civilian population in the US. Demographic characteristics, including immunization history and race and ethnicity were self-reported and collected by trained interviewers during a home interview. Participants provided self-collected cervicovaginal swab specimens that were evaluated by a polymerase chain reaction test and followed by type-specific hybridization. Detailed survey questionnaire, sample collection, and laboratory methods are available elsewhere.^2^

We estimated infection prevalence of HPV types 16 and 18 (HPV-16/18)—the oncogenic types covered by 4-valent vaccine—for 2 birth cohorts: women born in the 1980s (1980-1989) and the 1990s (1990-1998). To estimate HPV vaccination effectiveness, we estimated the prevalence of HPV-16/18 infection before vaccine introduction (2005-2006) and recently (2015-2016) from the NHANES data. Considering that the 1990s birth cohort was 26 years old or younger in 2015 and 2016, we limited the comparison to the 18- to 26-year-old age groups. In addition, we conducted a multivariable logistic regression to assess differences in estimated probabilities, with simultaneous adjustments made for the number of vaccine doses, age, race and ethnicity, country of birth, age at sexual debut, and the number of lifetime sex partners.

Data analyses were performed from November 2021 to February 2022 with SAS, version 9.4 (SAS Institute Inc) using the SAS PROC SURVEY procedures,^3^ which included weight, cluster, strata, and domain statements. Statistical significance was tested at 2-sided *P* < .05.

## Results

The study sample comprised 2698 women 18 to 26 years old (mean [SD] age, 21.5 [2.7] years; 879 [32.6%] White individuals). The prevalence of HPV-16/18 among participants born in the 1990s was statistically significantly lower (5.6%; 95% CI, 4.0%-7.2%) than those born in the 1980s (12.5%; 95% CI, 10.2%-14.7%; Figure, A). During the 2015−2016 cycle, 55% of women 18 to 20 years old, 52% of those 21 to 23 years old, and 50% of those 24 to 26 years old had received 1 or more doses of HPV vaccine. Among women 18 to 26 years old, HPV-16/18 prevalence before vaccination introduction (2005-2006 cycle) was 15.2% (95% CI, 11.2%-19.1%). In the recent (2015-2016) period, this percentage had declined to 3.3% (95% CI, 1.3%-5.3%) overall—5.1% (95% CI, 1.2%-9.1%) among unvaccinated and 1.0% (95% CI, 0.0%-2.5%) among vaccinated groups (Figure, B and C). Prevalence of HPV-16/18 infection in the recent (2015-2016) period was 0% among unvaccinated and vaccinated women 18 to 20 years old.

**Figure.**
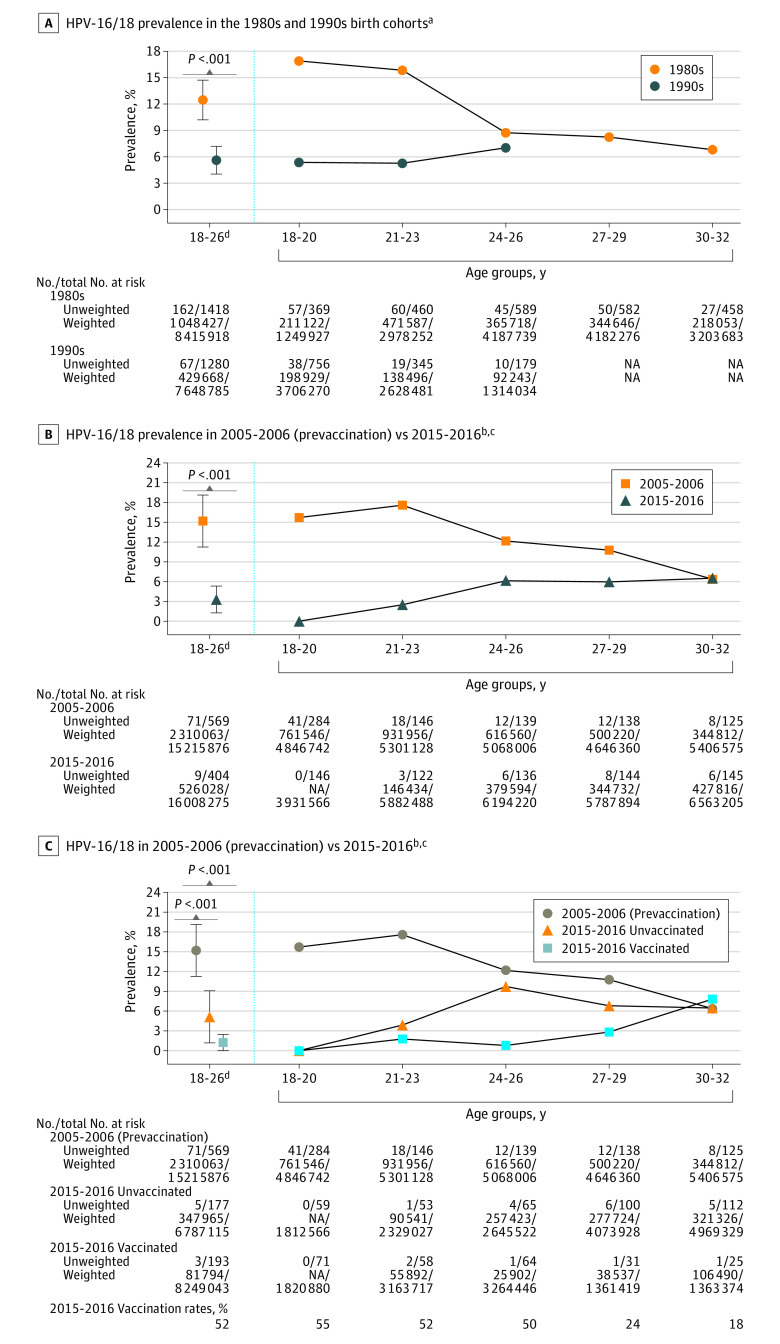
Prevalence Estimates of HPV-16/18 Infection Among Women Born in the 1980s and 1990s and During the Prevaccination Period (2005-2006) vs the Recent Vaccination Period (2015-2016), Overall and by HPV Vaccination Status Error bars indicate 95% CI. Nationally representative estimates for prevalence and the representative population counts were computed using the National Health and Nutrition Examination Survey sampling weights. HPV-16/18 indicates human papillomavirus, type 16 or 18. ^a^The final analytical sample for panel A consisted of women 18-32 years old born in the 1980s (n = 2458) and 1990s (n = 1280). ^b^The analytic sample for 2005-2006 and 2015-2016 comprised 832 and 693 women, respectively. ^c^The HPV vaccination rate in 2015-2016 was 52% among women 18-26 years old (18-20 y, 55%; 21-23 y, 52%; 24-26 y, 50%; 27-29 y, 24%; 30-32 y, 18%). ^d^Survey weight-adjusted Wald F test was used to compare prevalence estimates.

The estimated probability of HPV-16/18 infection was 54% lower for women born in the 1990s (6.3%; 95% CI, 5.7%-6.9%) than in the 1980s (13.6%; 95% CI, 12.7%-14.4%; Table). Similarly, the estimated probability was 78% lower overall during the 2015−2016 cycle (3.5%; 95% CI, 2.9%-4.1%) compared with the 2005−2006 cycle (15.7%; 95% CI, 14.0%-17.4%); more specifically, 60% lower for the unvaccinated (6.3%; 95% CI, 5.0%-7.4%) and 92% lower for the vaccinated (1.2%; 95% CI, 1.0%-1.3%). The estimated probabilities were lower for the 1990s (vs 1980s) birth cohort and for the recent (2015-2016) vs the prevaccination (2005-2006) period across race and ethnicity, lifetime number of sex partners, and country of birth categories.

**Table.** Differences in Estimated Probabilities of HPV-16/18 Infection Among Women Born in the 1980s vs 1990s and Prevaccination (2005-2006) vs Recent Vaccination (2015-2016) Periods, NHANES

Characteristic	Estimated probability of infection, % (95% CI)^a^					
	Born in 1980s	Born in 1990s	Difference^b^	Prevaccination, 2005-2006	Recent, 2015-2016	Difference^c^
Study population	13.6 (12.7-14.4)	6.3 (5.7-6.9)	7.3 (6.2-8.3)	15.7 (14.0-17.4)	3.5 (2.9-4.1)	12.2 (10.4-14.0)
HPV vaccine doses						
0 (unvaccinated)	14.8 (13.9-15.8)	9.1 (8.2-11.6)	5.7 (4.4-5.9)	15.7 (14.0-17.4)	6.3 (5.0-7.4)	9.5 (7.4-11.5)
≥1	6.0 (5.2-6.7)	3.2 (2.9-3.6)	2.7 (1.9-3.6)	NA	1.2 (1.0-1.4)	14.5 (12.8-16.2)
Race and ethnicity						
Hispanic	7.5 (6.6-8.3)	3.0 (2.7-3.3)	4.5 (3.6-5.4)	4.2 (3.1-5.4)	1.2 (0.9-1.5)	3.0 (1.8-4.3)
Non-Hispanic Black	17.2 (16.0-18.4)	7.7 (7.0-8.5)	9.4 (8.0-10.9)	17.3 (15.5-19.2)	4.9 (3.8-5.9)	12.5 (10.4-14.6)
Non-Hispanic White	13.5 (12.5-14.4)	6.0 (5.2-6.7)	7.5 (6.2-8.8)	17.3 (15.0-19.5)	3.6 (2.7-4.4)	13.7 (11.3-16.1)
Other^d^	22.0 (17.5-26.5)	12.9 (10.0-15.8)	9.1 (3.7-14.5)	20.2 (9.2-31.2)	5.5 (3.6-7.3)	14.7 (3.6-25.9)
Lifetime sex partners, No.						
0-1	2.1 (1.8-2.4)	0.8 (0.7-0.9)	1.3 (1.0-1.5)	2.4 (1.8-3.0)	0.5 (0.4-0.6)	1.9 (1.2-2.5)
2-5	12.8 (11.9-13.8)	6.0 (5.5-6.5)	6.9 (5.8-8.0)	17.2 (15.1-19.4)	3.9 (3.2-4.6)	13.3 (11.0-15.6)
≥6	18.7 (17.5-19.9)	9.7 (8.6-10.9)	9.0 (7.2-10.8)	21.7 (20.3-23.0)	4.5 (3.4-5.6)	17.2 (15.4-18.9)
Country of birth						
US	14.1 (13.2-15.0)	6.5 (5.8-7.1)	7.7 (6.5-8.8)	16.3 (14.4-18.2)	3.2 (2.6-3.8)	13.1 (11.1-15.0)
Not US	10.1 (7.9-12.4)	5.1 (4.0-6.2)	5.0 (2.6-7.5)	11.8 (6.1-17.5)	5.4 (2.9-7.9)	6.4 (0.1-12.7)

Abbreviations: HPV-16/18, human papillomavirus, type 16 or 18; NA, not applicable; NHANES, US National Health and Nutrition Examination Survey.

^a^ Model was simultaneously adjusted for the variables shown as well as for age and age at sexual debut as linear terms (sample limited to women 18-26 years old because those born in the 1990s were ≤26 years in 2015-2016).

^b^ Differences in estimated probabilities reflect reduction in estimated probabilities from the 1980s to the 1990s and from 2005-2006 to 2015-2016.

^c^ Difference compared with 2005-2006 (prevaccination).

^d^ Includes NHANES predefined categories: non-Hispanic American Indian/American Natives only, non-Hispanic American Indian/American Natives and any other group, other single race, and multiple races.

## Discussion

These study findings suggest that HPV vaccination was associated with a reduction in HPV-16/18 infection prevalence among a recent birth cohort of vaccinated and unvaccinated 18- to 26-year-old women. A larger decline in the prevalence of HPV-16/18 infection among 18- to 20-year-old women during the 2015−2016 time period may reflect greater direct and herd protection from broader HPV vaccination coverage.

These findings are consistent with a recent study demonstrating HPV vaccination effectiveness.^4^ Furthermore, this study provides a birth cohort perspective and suggests a change in the age distribution of HPV-16/18 prevalence. The study limitations were the use of self-reported HPV vaccination status and exclusion of HPV types not covered by the vaccine.

Historically in the US, HPV infection prevalence among women has followed a log-normal distribution pattern, with the peak observed among young age groups.^5^ This foundational concept may need to be reevaluated for HPV-16/18 infection, given the recent peak shift that we observed.

## References

[1] Markowitz LE, Dunne EF, Saraiya M, Lawson HW, Chesson H, Unger ER; Centers for Disease Control and Prevention (CDC); Advisory Committee on Immunization Practices (ACIP). Quadrivalent human papillomavirus vaccine: recommendations of the Advisory Committee on Immunization Practices (ACIP). MMWR Recomm Rep. 2007;56(RR-2):1-24. 17380109

[2] National Center for Health Statistics, US Centers for Disease Control and Prevention. National Health and Nutrition Examination Survey: questionnaires, datasets, and related documentation. Accessed July 21, 2022. https://wwwn.cdc.gov/nchs/nhanes/Default.aspx

[3] SAS Survey Analysis. Introduction to Survey Sampling and Analysis Procedures: the Survey Procedures. Accessed July 21, 2022. https://documentation.sas.com/doc/en/pgmsascdc/9.4_3.3/statug/statug_introsamp_sect002.htm

[4] Rosenblum HG, Lewis RM, Gargano JW, Querec TD, Unger ER, Markowitz LE. Human papillomavirus vaccine impact and effectiveness through 12 years after vaccine introduction in the United States, 2003 to 2018. Ann Intern Med. 2022. doi:10.7326/M21-3798 35576590 PMC11614147

[5] Schiffman M, Wentzensen N. Human papillomavirus infection and the multistage carcinogenesis of cervical cancer. Cancer Epidemiol Biomarkers Prev. 2013;22(4):553-560. doi:10.1158/1055-9965.EPI-12-1406 23549399 PMC3711590

